# Associations Between Severe Obesity and Depression: Results From the National Health and Nutrition Examination Survey, 2005-2006

**Published:** 2011-04-15

**Authors:** Arlene M. Keddie

**Affiliations:** Public Health and Health Education Programs, School of Nursing and Health Studies, Northern Illinois University

## Abstract

**Introduction:**

My objectives were to investigate the association between obesity and depression in a representative sample of American adults, investigate sex and severity of obesity as modifiers of the association between depression and body mass index, determine whether large waist circumference is associated with depression, and explore whether specific health behaviors and poor physical health are possible mediators of the association between obesity and depression, if found.

**Methods:**

The sample consisted of 3,599 nonpregnant adults aged 20 years or older from the National Health and Nutrition Examination Survey, 2005-2006. I operationalized obesity as body mass index (BMI) and waist circumference from the anthropometric measurements of participants and current depression from Patient Health Questionnaire (PHQ-9) scores. I ran logistic regression models with depression as the dependent variable.

**Results:**

In unadjusted analyses, large waist circumference (≥88 cm for women and ≥102 cm for men) and class III obesity (BMI ≥40 kg/m^2^) were associated with higher prevalence of depression in women only. All of these associations dramatically weakened after adjusting for demographic factors, self-rated health status, and number of chronic conditions.

**Conclusion:**

These findings support an association between depression and obesity in women who are severely obese. Future studies should investigate poor physical health as a possible mediator of the association between obesity and depression in this population of women.

## Introduction

Although the effects of obesity on physical health have been well documented (1), the consequences for mental health are less certain. If obesity and depression are causally related, one may help to perpetuate the other, increasing the risks for negative health outcomes beyond either of these conditions alone (2). Previous research on associations between depression and obesity has produced inconsistent results. Some studies have reported no association (3), some have reported positive associations (4-6), and some have reported negative associations (7,8). Reviewers have speculated that these inconsistent results are possibly indicative of associations among specific subgroups of the obese (9).

One likely effect modifier is sex. Among studies that found variation by sex, some found a positive association between depression and obesity in women and no association in men (4,5,10), others found a positive association in women and a negative association in men (11), and still others found no association in women and a negative association in men (12). The severity and type of obesity are also factors by which the association may differ. Only a few population-based studies have investigated possible relationships between the severity of obesity and depression (5,13), and many researchers have exclusively used body mass index (BMI), rather than abdominal fat, as a means of determining obesity (4,5,7,10-15).

Researchers have not adequately examined variables that may mediate this association (16). Possible mediating variables include poor physical health, known to be a long-term consequence of obesity (1) and to be associated with depression (16), and physical activity, which is protective against both obesity and depression (17,18). This study examines sex and severity of obesity as possible effect modifiers of the association between depression and obesity; examines the association by type of obesity (overall vs abdominal); and identifies demographic, behavioral, and health factors that may either confound or mediate these associations.

## Methods

### Study population

I conducted the analyses for this cross-sectional study by using data from the 2005-2006 National Health and Nutrition Examination Survey (NHANES), which is a stratified multistage probability sample of the civilian, noninstitutionalized US population that is conducted on an ongoing basis by the National Center for Health Statistics. The sampling frame consisted of all US counties, on the basis of the 2000 US census and associated estimates and projections. NHANES 2005-2006 oversampled African Americans, Mexican Americans, adolescents, and people with low income and who were aged 60 years or older. Details of the NHANES multistage sampling procedure are available elsewhere (19).

This study focused entirely on adults aged 20 years or older, of which there were 4,979 who had been interviewed during this 2-year period. Because indicators of obesity and depression can be influenced by pregnancy status, I excluded 440 women who were pregnant or whose pregnancy status was unknown, leaving 4,539 nonpregnant adults over age 20. Of these, 940 were excluded because of missing variables (BMI, waist circumference, depression screener score, or any of the covariates). A final sample of 3,599 remained (79% of those meeting inclusion criteria).

The 21% of eligible participants who were excluded were more likely to be less educated, racial/ethnic minorities, unmarried, older, physically inactive, to have lower income, and to report poor or fair health than those included in the study. However, no significant differences were found between the 2 groups by sex, smoking status, alcohol consumption, or number of chronic conditions; among those for whom data were available, neither were there significant differences by depression status, BMI, or waist circumference.

### Measurements

The Patient Health Questionnaire (PHQ-9) contains 9 questions that were used as a depression screener in NHANES 2005-2006. These are based on the 9 signs and symptoms for depression listed in the *Diagnostic and Statistical Manual of Mental Disorders, 4th edition* (DSM-IV). Responses to these 9 questions were on a 4-point Likert scale of 0 to 3, indicating that the participant experienced the symptom "not at all," "on several days," "on more than half the days," or "nearly every day" during the past 2 weeks. A 10th question assessed the degree of impairment these symptoms caused in the participant's daily life, again on a 4-point scale, from no impairment to extreme impairment (20).

I included in these analyses only participants who had completed the entire PHQ-9, since it was impossible to determine the true PHQ-9 score of those who did not. I operationalized depression as a dichotomous dependent variable. To be considered "depressed," a participant had to score 10 or more, indicating a moderate to severe level of depressive symptoms. Tested against a structured mental health professional interview, a PHQ-9 score of at least 10 had a sensitivity and specificity of 88% for a clinical diagnosis of major depression (21). This group included participants with major depressive disorder (MDD). To be considered to have MDD, a participant had to indicate that he or she experienced at least 5 of the 9 symptoms "on more than half the days" during the past 2 weeks. One of the 5 had to include "little interest in doing things" or "feeling down, depressed, or hopeless." Contemplation of suicide was included if positively indicated at all (20). Because current MDD was rare (2.2% weighted prevalence), to maximize statistical power a PHQ-9 score of 10 or more (5.1% weighted prevalence) was used in these analyses. The same analyses were repeated with MDD. The results were similar (analyses not shown).

I used 2 indicators of obesity in this study. The first was BMI, consisting of weight in kilograms divided by height in meters squared. Waist circumference was also used as an indicator of intraabdominal fat. In NHANES, trained examiners measured weight in pounds and converted the measurement to the nearest 0.1 kg by using an automated system. They measured standing height to the nearest 0.1 cm for all participants who were able to stand unassisted and waist circumference to the nearest 0.1 cm at the end of a normal expiration at the level of the iliac crest (22).

I split BMI into the 6 categories recommended by the National Heart, Lung, and Blood Institute: underweight (<18.5 kg/m^2^), normal weight (18.5-24.9 kg/m^2^), overweight (25.0-29.9 kg/m^2^), class I obesity (30.0-34.9 kg/m^2^), class II obesity (35.0-39.9 kg/m^2^), and class III obesity (≥40.0 kg/m^2^) (23). For all BMI comparisons, the normal weight group was used as the reference. I dichotomized waist circumference into high-risk and low-risk groups, on the basis of the sex-specific cut points of 88 cm or more for women and 102 cm or more for men, recommended by the National Institutes of Health (23). Women with a waist circumference of less than 88 cm and men with a waist circumference of less than 102 cm were used as the reference groups.

I made a priori choices of covariates based on a review of the literature (9). I included the following potential demographic confounders: age, sex, race/ethnicity, education, annual household income, and marital status. I categorized age into 20 to 39 years, 40 to 59 years, and 60 years or older (reference group). Non-Hispanic whites were the reference group to which non-Hispanic blacks, Hispanics, and others were compared. I dichotomized educational level into the reference group of college-educated participants and those with less than a 4-year college degree. NHANES collected data on annual family household income in categorical form. I collapsed these data into 4 categories: $55,000 or more (reference group), $35,000 to $54,999, $20,000 to $34,999, and less than $20,000. I kept marital status in the 6 original categories in which the data were collected: married (reference group), widowed, divorced, separated, never married, and living with partner.

I included 3 health behaviors (smoking status, alcohol consumption, and physical activity) and 2 health indicators (self-rated health status and number of chronic conditions) in separate models as possible mediators. I dichotomized smoking status into current smokers and nonsmokers (reference group); categorized alcohol consumption into abstainers or very light drinkers, moderate drinkers (reference group), and binge drinkers; and split physical activity into participants who reported engaging in at least 10 minutes of vigorous or moderate leisure-time aerobic activity during the past 30 days (reference group) and those who did not. I compared the self-reported health status ratings of "excellent, very good, or good" (reference group) to "fair or poor." I created an ordinal variable with 4 categories on the basis of the number of chronic conditions a person reported and separately compared participants with 1, 2, or 3 or more of these conditions to those who reported none. These chronic conditions were arthritis, heart disease, stroke, emphysema, chronic bronchitis, a liver condition, a thyroid problem, and cancer.

### Statistical analysis

I weighted all analyses using STATA 10 (StataCorp LP, College Station, Texas) survey commands to account for oversampling of people aged 60 years or older, African Americans, and Mexican Americans; nonresponse; and the design of the sample (clustering and stratification). I calculated the prevalence of depression across sex and BMI or waist circumference categories, and ran logistic regression models with depression as the dependent variable and obesity as the independent variable. Because of the high correlation between BMI and waist circumference (Pearson's correlation coefficient = 0.88), I did not put them into the same model. I ran 7 models, each with BMI or waist circumference as the main independent variable of interest: an unadjusted model; and models controlling for demographic covariates; both demographic and behavioral covariates; demographic and health covariates; only health covariates; and, to distinguish between the effects of number of chronic conditions and self-rated health, 2 more models in which I controlled for each of these separately. I conducted all analyses separately for men and women. Given the prevalence of depression and obesity within each sex and the size of the eligible sample, power calculations indicated that a significant odds ratio (OR) of 1.84 among men and 1.75 among women could be detected with the level of type I error set at *P* = .05 and type II error at *P* = .20.

## Results

The proportion of women in the sample who were older, had less income, had more chronic conditions, were underweight or obese, had a higher than optimal waist circumference, and were depressed was higher than that of the men (Table 1). Years of education, recreational aerobic activity, and self-rated health status were similar between the sexes. A total of 212 participants had a PHQ-9 score of at least 10: 90 men and 122 women, representing 4% of men and 6% of women, respectively. These estimates are within the range found in the literature for current depression (24) and follow the pattern found in studies of depression by sex in developed countries (25).

Women with a large waist circumference had almost double the prevalence of depression of those with a small waist circumference (Table 2). Prevalence of depression fluctuated but was similar among normal-weight women, overweight women, and women with class I obesity. Prevalence of depression began to rise among women with class II obesity and rose sharply among women with class III obesity. Among men, no significant differences were found among the BMI classifications. Only 64 people in the sample were underweight, 25 men and 39 women. Because only 1 underweight woman and 1 underweight man were depressed, this BMI category was eliminated from the multivariate analyses shown in Table 3.

Women with a BMI of 40 kg/m^2^ or more had more than 4 times the odds of being depressed as women with a BMI between 18.5 kg/m^2^ and 24.9 kg/m^2^ (Table 3). This association was reduced but remained significant (OR = 3.05) after controlling for demographic variables, increased slightly (OR = 3.24) when behavioral variables were included, and then lost significance, falling slightly to just over 2 when the model was adjusted for demographic factors and health. Women with a large waist circumference were 1.8 times as likely to be depressed as women with a small waist circumference. This association lost significance after adjusting for demographic variables and for all other models.

To determine which of the 2 health variables explained more of the association between depression and obesity in women, I examined them separately. Although neither was sufficient to account for all of the association among women with class III obesity, together they reduced the OR substantially, even when demographic factors were left out of the model. Self-rated health accounted for more of the association than did number of chronic conditions. No significant differences were found for prevalence of depression by BMI or waist circumference among men.

## Discussion

This study has several strengths not found in many previous cross-sectional studies of depression and obesity: depressive symptoms were measured by using a clinically valid instrument based on DSM-IV criteria; measured anthropometry was used to estimate BMI and waist circumference; a large, recent representative sample with a wide age range and high response rate was used; a range of covariates was adjusted for; obesity was operationalized in more than 1 form; and the effects of health behaviors and physical health as possible mediators were examined.

This study also has several limitations. One limitation is low statistical power. Although the total sample size was large, current depression, for which the PHQ-9 is a screening tool, is rare, particularly in men, which limited my ability to find significant associations for low ORs. Since the prevalence of depression was lower among men in general, this could have at least partially accounted for the lack of significance in the waist circumference models, in which point estimates were similar for men and women; however, none of the point estimates for associations among severely obese men came close to those found among women with class III obesity.

A second limitation is the cross-sectional design of the study, which did not allow me to determine whether depression preceded obesity or vice versa, a necessary criterion for determining causality. A final limitation is the possibility of selection bias due to the exclusion of participants with missing data. Nevertheless, this study was consistent with others that found associations only among women (4,5,10). These results are consistent with previous analyses of NHANES III, NHANES 2005-2006, and clinical samples of obese adults, in finding that adults with class III obesity have a higher prevalence of depression than adults in other BMI categories (5,13,26).

Adjustment for demographic factors reduced the odds of depression in women with class III obesity compared with women who were normal weight. Low income and low education accounted for most of this drop in point estimates. Further research should focus on these vulnerable groups.

Adjustment for 3 health behaviors did not substantially change the ORs, but adjustment for the number of chronic conditions and self-rated health further reduced the strength of the association among women with class III obesity by 50% and resulted in a loss of significance. The effect of physical health was also seen when the demographic factors were removed from the model, producing an OR that was not very different from that of the full demographic and health model. The combined effect of both self-rated health and number of chronic conditions was responsible for reducing this association substantially, although self-rated health had a stronger effect.

Because self-rated health is likely to be influenced by the mental state of the study participant, at least 2 explanations for these results are possible: either poor health is a true mediator of obesity and depression, or a poor health self-rating is at least partially a consequence of being in a depressed state and is independently a consequence of obesity. If poor health is a true mediator, a possibility is that earlier obesity resulted in poor physical health, which increased the likelihood of depression. Although previous depression could lead to ill health, it is less likely that poor physical health was a cause of obesity, rather than the other way around. The order of events cannot be determined in a cross-sectional study, but depression, severe obesity, and ill health appear to be strongly interconnected in women.

There are strengths and weaknesses in the use of these 2 health indicators. Self-rated health, although subjective, is strongly associated with future mortality (27). Number of chronic conditions, which appears to be more of a hard outcome, less influenced by the perceptions of the participant, is still only an approximation of health status, since the conditions included here ranged in seriousness from strokes to thyroid problems, which could have been under varying degrees of control.

Results of adjustment for physical health have varied in other studies. In a cross-sectional study by Jorm et al, an association between obesity and depression, which appeared to be entirely mediated by self-reported physical health, was found among women (4). However, in NHANES III, after controlling for physicians' health rating, the association between class III obesity and depression remained strong and significant (5). In their prospective study of an older American population, after adjusting for the presence of 2 or more chronic conditions and limitations for activities of daily living, Roberts et al found that the association between prior obesity and later depression was weakened from an OR of 2.06 to 1.79 (6). Vogelzangs et al confirmed these findings only among men in their cohort of older people, after controlling for number of chronic conditions, cardiovascular disease, and diabetes (28). In this study, the odds of depression among women with class III obesity was reduced from 4.29 to 2.13 after controlling for self-rated health and number of chronic conditions.

Future prospective studies should investigate whether and to what degree associations between obesity and depression are mediated by poor physical health, particularly in middle-aged and older people, and whether these associations occur only in severely obese women. If confirmed by other studies, clinicians should consider the high probability of depression in severely obese women, screen them for it, and modify their treatment plans accordingly.

## Figures and Tables

**Table 1:** Sample Characteristics, Stratified by Sex, National Health and Nutrition Examination Survey, 2005-2006 (N = 3,599)

Characteristic	Men (N = 1,871)	Women (N = 1,728)	*P* Value^a^

	No. (Weighted %)	No. (Weighted %)	
**Age, y**			
20-39	682 (40.2)	559 (33.7)	.002
40-59	604 (39.9)	613 (42.0)	
≥60	585 (19.9)	556 (24.3)	
**Race/ethnicity**			
Non-Hispanic white	983 (74.2)	888 (73.8)	.08
Non-Hispanic black	412 (10.1)	399 (11.2)	
Hispanic	421 (11.4)	368 (9.8)	
Others	55 (4.3)	73 (5.3)	
**Education**			
College-educated	385 (26.5)	371 (27.4)	.56
Less than a college degree	1,486 (73.5)	1,357 (72.6)	
**Annual family income, $**			
<20,000	417 (14.6)	420 (18.0)	.05
20,000-34,999	421 (18.7)	373 (18.3)	
35,000-54,999	386 (21.3)	350 (20.2)	
≥55,000	647 (45.4)	585 (43.4)	
**Marital status**			
Married	1,128 (61.7)	856 (55.5)	<.001
Widowed	76 (2.1)	221 (9.1)	
Divorced	163 (8.7)	216 (12.6)	
Separated	47 (2.3)	63 (2.5)	
Never married	293 (15.9)	259 (13.6)	
Living with partner	164 (9.3)	113 (6.8)	
**Smoking status**			
Current smoker	496 (27.6)	324 (20.8)	<.001
Nonsmoker	1,375 (72.4)	1,404 (79.2)	
**Alcohol consumption**			
Abstainer or very light drinker^b^	300 (14.0)	704 (33.6)	<.001
Moderate drinker^c^	1,165 (64.2)	934 (60.5)	
Binge drinker^d^	406 (21.8)	90 (5.9)	
**Recreational aerobic activity**			
Some^e^	1,192 (69.3)	1,089 (68.4)	.60
None^f^	679 (30.7)	639 (31.6)	
**Number of chronic conditions^g^**			
0	1,232 (69.0)	958 (55.7)	<.001
1	415 (22.6)	458 (27.4)	
2	151 (5.7)	209 (11.6)	
≥3	73 (2.8)	103 (5.4)	
**Self-rated health**			
Excellent, very good, or good	1,501 (85.3)	1,353 (84.0)	.35
Fair or poor	370 (14.7)	375 (16.0)	
**Body mass index, kg/m^2^**			
<18.5 (underweight)	25 (1.2)	39 (2.5)	<.001
18.5-24.9 (normal weight)	478 (25.5)	545 (35.9)	
25.0-29.9 (overweight)	773 (39.9)	473 (25.9)	
30.0-34.9 (class I obesity)	387 (21.5)	338 (17.6)	
35.0-39.9 (class II obesity)	132 (7.7)	197 (10.7)	
≥40.0 (class III obesity)	76 (4.2)	136 (7.4)	
**Waist circumference, cm**			
<88 for women or <102 for men	1,062 (55.8)	630 (40.9)	<.001
≥88 for women or ≥102 for men	809 (44.2)	1,098 (59.1)	
**Depression status**			
Depressed	90 (4.2)	122 (6.0)	.02
Not depressed	1,781 (95.8)	1,606 (94.0)	

a Pearson *χ* ^2^ comparison for men and women.

b Consumed  fewer than 12 alcoholic drinks in the previous year and never consumed 5 or more drinks at a time.

c Consumed at least 12 alcoholic drinks in the previous year but never consumed 5 or more drinks at a time.

d Consumed 5 or more drinks at a time.

e At least 10 minutes of vigorous or moderate leisure-time aerobic activity within the past month.

f No leisure-time aerobic activity within the past month.

g The sum of the number of the following chronic conditions: arthritis, heart disease, stroke, emphysema, chronic bronchitis, a liver condition, a thyroid problem, and cancer.

**Table 2:** Percentage Depressed, by Body Mass Index, Waist Circumference, and Sex, National Health and Nutrition Examination Survey, 2005-2006

**Characteristic**	Number Depressed/Total	Weighted %	*P* Value^a^
**Men (n = 1,871)**			
**Body mass index, kg/m^2^**			
<18.5 (underweight)	1/25	1.0	.24
18.5-24.9 (normal weight)^b^	23/478	4.2	
25.0-29.9 (overweight)	26/773	2.8	
30.0-34.9 (class I obesity)	28/387	6.1	
35.0-39.9 (class II obesity)	8/132	6.4	
≥40.0 (class III obesity)	4/76	5.5	
Total	90/1,817	4.2	
**Waist circumference**			
Small^c^ (<102 cm)	43/1,062	3.5	.29
Large (≥102 cm)	47/809	5.2	
**Women (n = 1,728)**			
**Body mass index, kg/m^2^**			
<18.5 (underweight)	1/39	1.3	<.001
18.5-24.9 (normal weight)^b^	33/545	4.9	
25.0-29.9 (overweight)	29/473	5.2	
30.0-34.9 (class I obesity)	22/338	4.8	
35.0-39.9 (class II obesity)	11/197	6.4	
≥40.0 (class III obesity)	26/136	18.2	
Total	122/1,728	6.0	
**Waist circumference**			
Small^c^ (<88 cm)	30/630	4.0	.01
Large (≥88 cm)	92/1,098	7.4	

a *P* values calculated by using Pearson *χ* ^2^ test.

b Referent group for comparison of prevalence of depression by body mass index category.

c Referent group for comparison of prevalence of depression by waist circumference category.

**Table 3:** Associations Between Obesity and Depression Among Women and Men, National Health and Nutrition Examination Survey, 2005-2006

Variables	Unadjusted	Demographic Model^a^	Demographic and Behavioral Model^b^	Demographic and Health Model^c^	Health Model^d^	No. of Chronic Conditions Only	Self-Rated Health Only

	OR (95% CI)	OR (95% CI)	OR (95% CI)	OR (95% CI)	OR (95% CI)	OR (95% CI)	OR (95% CI)
**Women**							
**Waist circumference,^e^ cm**							
<88	1 [Ref]	1 [Ref]	1 [Ref]	1 [Ref]	1 [Ref]	1 [Ref]	1 [Ref]
≥88	1.82 (1.10-3.01)	1.57 (0.92-2.69)	1.53 (0.90-2.58)	1.01 (0.59-1.71)	1.00 (0.66-1.54)	1.34 (0.79-2.30)	1.21 (0.78-1.87)
**Body mass index,^f^ kg/m^2^**							
18.5-24.9	1 [Ref]	1 [Ref]	1 [Ref]	1 [Ref]	1 [Ref]	1 [Ref]	1 [Ref]
25.0-29.9	1.06 (0.63-1.81)	0.97 (0.52-1.62)	1.04 (0.53-2.04)	0.82 (0.39-1.73)	0.81 (0.45-1.45)	0.86 (0.47-1.57)	0.93 (0.56-1.54)
30.0-34.9	0.98 (0.48-1.99)	0.79 (0.39-1.59)	0.83 (0.42-1.66)	0.53 (0.27-1.04)	0.58 (0.31-1.08)	0.80 (0.39-1.62)	0.65 (0.34-1.25)
35.0-39.9	1.32 (0.51-3.45)	0.98 (0.35-2.75)	1.03 (0.37-2.87)	0.54 (0.21-1.44)	0.71 (0.29-1.73)	1.00 (0.43-2.31)	0.81 (0.30-2.19)
≥40.0	4.29 (1.86-9.90)	3.05 (1.12-8.35)	3.24 (1.16-9.07)	2.02 (0.86-4.76)	2.13 (1.09-4.17)	3.43 (1.44-8.17)	2.35 (1.20-4.62)
**Men**							
**Waist circumference,^e^ cm**							
<102	1 [Ref]	1 [Ref]	1 [Ref]	1 [Ref]	1 [Ref]	1 [Ref]	1 [Ref]
≥102	1.50 (0.66-3.42)	1.63 (0.75-3.56)	1.63 (0.75-3.55)	1.17 (0.53-2.60)	0.98 (0.41-2.33)	1.21 (0.50-2.92)	1.09 (0.46-2.57)
**Body mass index,^f^ kg/m^2^**							
18.5-24.9	1 [Ref]	1 [Ref]	1 [Ref]	1 [Ref]	1 [Ref]	1 [Ref]	1 [Ref]
25.0-29.9	0.64 (0.25-1.69)	0.80 (0.33-1.91)	0.85 (0.36-1.99)	0.61 (0.24-1.54)	0.56 (0.21-1.50)	0.58 (0.22-1.55)	0.61 (0.23-1.59)
30.0-34.9	1.48 (0.47-4.67)	1.79 (0.59-5.43)	1.91 (0.59-6.16)	1.31 (0.41-4.20)	1.10 (0.34-3.57)	1.26 (0.40-3.98)	1.22 (0.38-3.90)
35.0-39.9	1.56 (0.44-5.57)	1.69 (0.52-5.46)	1.85 (0.56-6.05)	1.02 (0.29-3.62)	0.89 (0.22-3.64)	1.24 (0.34-4.56)	0.93 (0.23-3.83)
≥40.0	1.31 (0.27-6.40)	1.50 (0.35-6.49)	1.64 (0.41-6.48)	0.61 (0.14-2.74)	0.61 (0.13-2.84)	1.17 (0.21-6.56)	0.60 (0.14-2.57)

Abbreviations: OR, odds ratio; CI, confidence interval; ref, reference.

a Adjusted for race/ethnicity, education, income, marital status, and age.

b Adjusted for the same variables as the demographic model plus smoking, alcohol consumption, and leisure-time physical activity.

c Adjusted for the same variables as the demographic model plus self-rated health and number of chronic conditions.

d Adjusted for self-rated health and number of chronic conditions only.

e Small waist circumference for women, <88 cm; small waist circumference for men, <102 cm. Large waist circumference for women, ≥88 cm; large waist circumference for men, ≥102 cm.

f Body mass index category descriptions: less than 18.5 kg/m^2^, underweight; 18.5 to 24.9 kg/m^2^, normal weight; 25.0 to 29.9 kg/m^2^, overweight; 30.0 to 34.9 kg/m^2^, class I obesity; 35.0 to 39.9 kg/m^2^, class II obesity; 40 kg/m^2^ or more, class III obesity.
